# Loss of heterozygosity related to TMB and TNB may predict PFS for patients with SCLC received the first line setting

**DOI:** 10.1186/s12967-021-03019-6

**Published:** 2021-09-08

**Authors:** Chenyue Zhang, Kai Wang, Haiyong Wang

**Affiliations:** 1 Department of Integrated Therapy, Fudan University Shanghai Cancer Center, Shanghai Medical College, Shanghai, 200032 China; 2 Key Laboratory of Epigenetics and Oncology, the Research Center for Preclinical Medicine, Southwest Medical University, Luzhou, 646000 China; 3 Department of Internal Medicine Oncology, Shandong Cancer Hospital and Institute, Shandong First Medical University and Shandong Academy of Medical Sciences, Number 440, Ji Yan Road, Jinan, 250117 China

## **To the Editor,**

Genes have two alleles or copies, with one inherited from each parent. The loss of one of these gene copies, termed as loss of heterozygosity (LOH), is one of the most common genetic alterations in cancer [1]. LOH has been playing a pivotal role in cancer development [2]. For instance, LOH was found to be involved in the relapse of acute lymphoblastic leukemia [3]. LOH of human leukocyte antigen (HLA) alleles hampered the ability of major histocompatibility complex to present neoantigens, thus implicating in resistance to immune checkpoint blockade (ICB) therapy [4]. Therefore, expounding the relationship between LOH and cancer will be very helpful to guide the accurate treatment.

Small cell lung cancer (SCLC) is featured by rapid growth and tendency to metastasize, with grim prognosis and high relapse rate [5]. However, the landscape of LOH and its impact on prognosis and relapse has remained largely unknown in SCLC. Moreover, the association between LOH and immunological features has never been studied in SCLC.

**Fig. 1:**
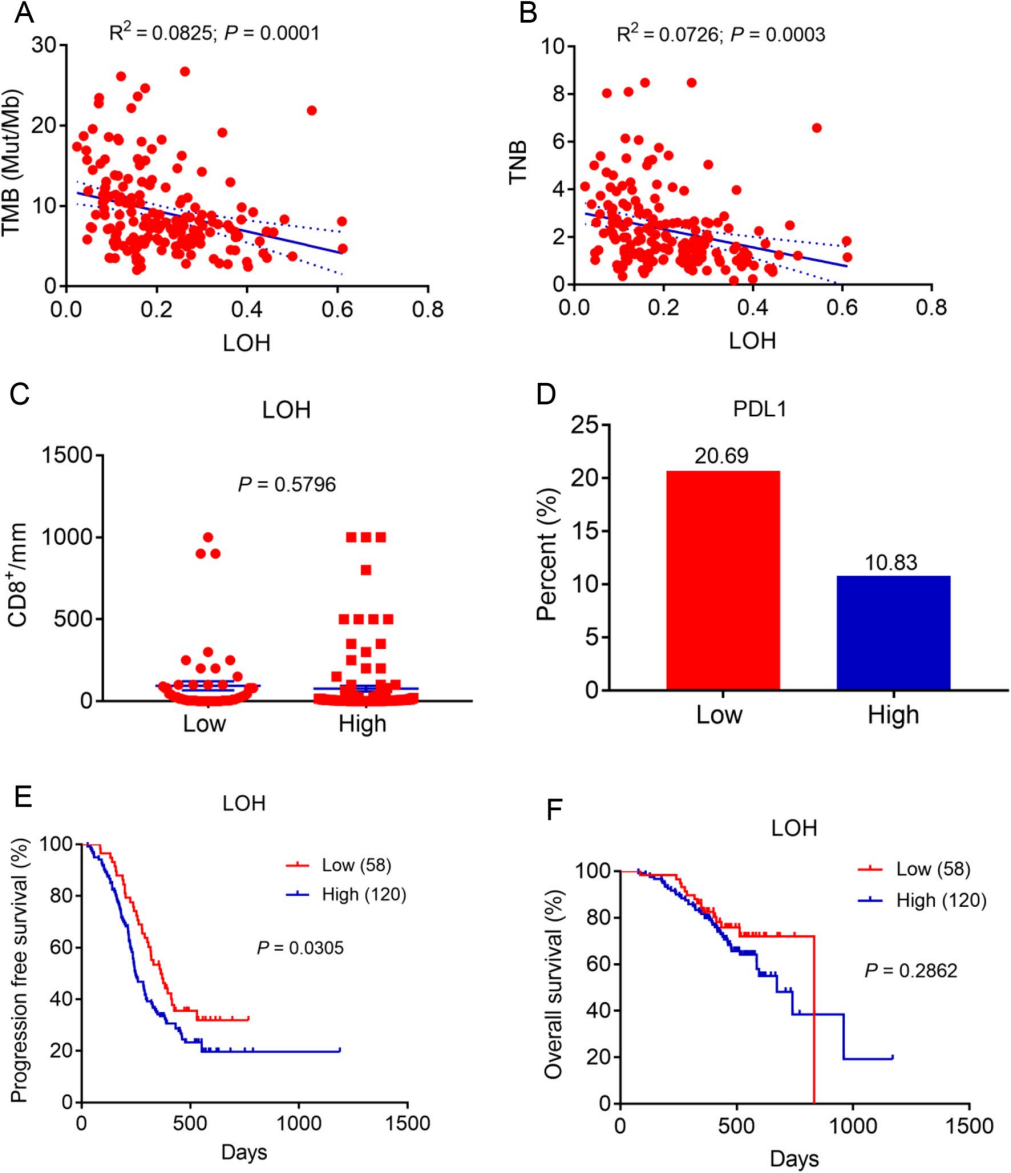
The association of LOH with immune-related markers and the impact of LOH on survival. **A** The association between LOH and TMB for SCLC patients. **B** The association between LOH and TNB for SCLC patients. **C** The difference in CD8  +  TIL infiltration between low-LOH and high-LOH in SCLC patients. **D** The difference in positive PD-L1 expression between low-LOH and high-LOH in SCLC patients. **E** The effect of LOH on PFS in SCLC patients. The cutoff for LOH is determined by X-tile. **F** The effect of LOH on OS in SCLC patients

A total of 178 histologically confirmed SCLC patients were collected from Shandong Cancer Hospital and Institute. This study was approved by the Ethics Committee of Shandong Cancer Hospital and Institute. All included patients in this study offered written informed consent. Whole-exome-sequencing (WES) analyses were performed to detect the of the tumor mutational burden (TMB) and tumor neoantigen burden (TNB) for SCLC patients. We have demonstrated that SCLC patients with higher LOH were associated with lower tumor mutational burden (TMB) (R square  =  0.0825, *P * =  0.0001; Fig. 1A). Similarly, LOH was found to be negatively associated with lower tumor neoantigen burden (TNB) (R square  =  0.0726, *P * =  0.0003; Fig. 1B). Since CD8  +  T cells are the body’s main immunological barrier against cancer and PD-L1 expression has reported to be a biomarker for immunotherapy, we next analyzed the association between CD8  +  T cell infiltration, PD-L1 expression and LOH in SCLC. CD8  +  TIL density and PD-L1 expression was measured using immunohistochemistry. In addition, X-tile software was applied to determine the optimal cutoff of LOH to differentiate progression free survival (PFS) (endup point for PFS: 15th, December, 2020) [6]. LOH was divided low and high in light of the optimal cutoff. The results showed that there was no significant difference in CD8  +  T cell infiltration between low-LOH and high-LOH SCLC patients (*P * =  0.5796; Fig. 1C). SCLC patients with low LOH had numerically higher PD-L1 positive expression than those with high LOH (20.69% versus 10.83%; Fig. 1D). Importantly, in the LOH-low cohort, PFS was significantly prolonged compared with that in LOH-high cohort (*P * =  0.0305; Fig. 1E). Moreover, multivariate Cox regression analyses were further conducted to evaluate the prognostic factors on PFS. We have found that LOH remains to be an independent factor for predicting PFS even after adjusting for factors including age, sex, smoking, family history and stage (HR, 1.574; 95% CI 1.033–2.398; *P * =  0.035; Additional file 1: Table S1). Additionally, there was no significant difference in overall survival (OS) (endup point for OS: 26th, November, 2020) between LOH-low and LOH-high cohort (*P * =  0.2862; Fig. 1F).

To the best of our knowledge, we are the first to analyze the association between immune-related markers including TMB, TNB, CD8  +  TIL, PD-L1 and LOH for patients with SCLC. We not only demonstrated the negative association between LOH and TMB, TNB in SCLC, but also revealed that low LOH is associated with prolonged PFS. We concluded that LOH may predict PFS by negatively affecting TMB and TNB in SCLC. Our finding suggests that LOH is a very valuable benchmark that predicts PFS in SCLC. Undeniably, more clinical and translational researches are warranted for confirmation of LOH’s role in SCLC.

## Supplementary Information

**Additional file 1: Table S1.** Multivariate Cox regression analyses to evaluate the prognostic factors for PFS.

## Data Availability

The data are available from the corresponding authors upon reasonable request.
