# Supplementary material for: Loss of heterozygosity related to TMB and TNB may predict PFS for patients with SCLC received the first line setting
Source: J Transl Med. 2021 Sep 8;19:385. doi: 10.1186/s12967-021-03019-6 (PMC8425073; doi:10.1186/s12967-021-03019-6)
Supplement: Supplementary file 1 — Additional file 1: Table S1. Multivariate Cox regression analyses to evaluate the prognostic factors for PFS. [file 12967_2021_3019_MOESM1_ESM.docx]

**Supplementary Table 1. Multivariate Cox regression analyses to evaluate the prognostic factors for PFS**

|  | **Multivariate analyses** | | |
| --- | --- | --- | --- |
| **Variable** | **HR** | **95% CI** | ***P*** |
| **Age** |  |  |  |
| < 65 | Reference |  |  |
| ≥65 | 0.828 | 0.562-1.219 | 0.338 |
| **Sex** |  |  |  |
| Female | Reference |  |  |
| Male | 1.128 | 0.640-1.990 | 0.676 |
| **Smoking** |  |  |  |
| Yes | Reference |  |  |
| No | 0.981 | 0.580-1.659 | 0.942 |
| **Family history** |  |  |  |
| Yes | Reference |  |  |
| No | 0.607 | 0.400-0.921 | 0.019 |
| Unknown | 0.472 | 0.206-1.080 | 0.075 |
| **Stage** |  |  |  |
| Limited | Reference |  |  |
| Extensive | 2.138 | 1.485-3.078 | < 0.0001 |
| **LOH** |  |  |  |
| Low | Reference |  |  |
| High | 1.574 | 1.033-2.398 | 0.035 |
